# Editorial: Advanced deep learning approaches for medical neuroimaging data with limitation

**DOI:** 10.3389/fncom.2023.1272448

**Published:** 2023-08-29

**Authors:** Zhiri Tang, Ming Li, Ruihan Hu, Kapal Dev

**Affiliations:** ^1^Department of Computer Science, City University of Hong Kong, Kowloon, Hong Kong SAR, China; ^2^Department of Industrial and Systems Engineering, The Hong Kong Polytechnic University, Kowloon, Hong Kong SAR, China; ^3^School of Software, Harbin Institute of Technology, Harbin, China; ^4^Department of Computer Science, Munster Technological University, Cork, Ireland

**Keywords:** neuroimaging, analysis of insufficient data, brain diseases, data processing, data augmentation, neuroimaging application

This Research Topic collects the latest works focusing on approaches and analysis for medical neuroimaging data with limitations. With the increasing development of deep learning and artificial intelligence, it also attracts attention from researchers in medical and clinical fields (Shen et al., 2017). Adopting deep learning technology in processing medical images and signals and benefiting from the powerful feature extraction ability of convolutional neural networks, deep learning-based artificial medicine has achieved great success in the past few years (Litjens et al., 2017).

However, owing to the difference between natural and medical images, in other words, some specific characteristics of medical images, adopting deep learning to process medical images directly may face numerous problems. Particularly, medical imaging, including neuroimaging data, may acquire data with few annotations, low signal-to-noise ratio, or small experimental samples, resulting in serious performance degradation during data processing (Tajbakhsh et al., 2020). Hence, how to solve these issues and improve the data analysis performance is still a challenge.

In recent years, some works have emerged toward these issues in medical neuroimaging data, such as semi-supervised learning (Wang et al., 2020), weakly supervised learning (Zhang et al., 2023), unsupervised learning (Zhao et al., 2019), transfer learning (Raghu et al., 2019), and so on. In this Research Topic, there are also some feasible ways for medical neuroimaging data with limitations.

In the first article of this Research Topic (Ma et al.), a Soft-DTW-based single-subject short-distance event-related potential averaging method is introduced for the low signal-to-noise ratio and feature extraction issues of N400 data. Specifically, N400 data is a time-domain EEG feature, which is thought to reflect the information processing ways of human brains. Leveraging the benefits of the differentiable and efficient Soft-DTW loss function, this work also involves performing partial Soft-DTW averaging within a single-subject range, effectively exploiting the advantages of the DTW distance. Furthermore, a Transformer-based recognition model incorporating location coding and a self-attention mechanism for contextual information is also presented. Finally, Softmax is selected as the classifier. Numerous experiments on the N400 public dataset demonstrate the performance of the proposed model and averaging method. These results validate the capabilities of the proposed approach in effectively handling ERP data and improving classification accuracy.

In the second article of this Research Topic (Wang et al.), some machine learning methods are adopted for forecasting stroke recurrence within 1 year in patients who have experienced acute ischemic stroke (AIS). Recurrent strokes contribute to approximately 25–30% of all preventable strokes, making the development of an accurate predictive tool crucial for identifying high-risk patients and implementing timely preventive measures. Univariate and multivariate logistic regression (LR) analyses are employed to identify potential risk factors associated with stroke recurrence. The dataset is randomly split into a training and a test set with a 7:3 ratio. Subsequently, six machine learning models are established, including random forest (RF), Naive Bayes model (NBC), decision tree (DT), extreme gradient boosting (XGB), gradient boosting machine (GBM), and LR. To determine the model with the most robust prediction performance, 10-fold cross-validation, receiver operating characteristic (ROC) curves, and SHapley Additive exPlanations (SHAP) are utilized. Finally, a user-friendly web calculator is built for better visualization and interpretation.

In the third article of this Research Topic (Liu et al.), federated learning (FL) is adopted for multiple sclerosis (MS) lesion segmentation. Specifically, FL aims to be widely applied in the medical image analysis field, which could learn from multi-site clients and keep the privacy of local sites. FL has shown promising applications in various domains, but its potential in neuroimage analysis tasks, like lesion segmentation in MS, is yet to be fully optimized. The challenges arise from the variability in lesion characteristics caused by using different scanners and acquisition parameters across multiple data sources. In this work, learnable weights are allocated according to the performance of each site. Furthermore, a weighted segmentation loss function is also introduced based on the lesion volume of each site. Two datasets, including one public and one clinical dataset, are adopted to verify the segmentation performance of the proposed framework.

In the fourth article of this Research Topic (Hu et al.), the authors focus on medical prediction from missing data. Unlike natural image datasets, missing data is a general issue in the medical image field. In the current research, the uncertainty associated with imputation can lead the model to overfit the observed data distribution, subsequently impacting its generalization performance negatively. While R-Drop is a potent regularization method for training deep neural networks, it cannot distinguish between positive and negative samples, thereby hindering the model's capacity to learn robust representations. In this work, a modified R-Drop method with negative regularization is proposed to boost the generalization and performance of medical image processing. The modified framework introduces a deliberate inconsistency between the output distributions of positive and negative samples. Notably, it also introduces a max-minus negative sampling technique that enhances model diversity by subtracting the mini-batch values from the maximum in-batch values to create negative samples. Three real-world medical prediction datasets, including both missing and complete data, are involved in the experimental parts.

Concerning the above papers in this Research Topic, they provide some novel ideas and approaches for medical neuroimaging data with limitations. We hope that the readers can be inspired by these state-of-the-art works and introduce more effective and powerful ways in neuroscience.

## Author contributions

ZT: Writing—original draft. ML: Writing—review and editing. RH: Writing—review and editing. KD: Writing—review and editing.
